# Displaced People Health, a Challenge for Epidemiology and Public Health

**DOI:** 10.3389/ijph.2024.1607580

**Published:** 2024-07-08

**Authors:** Rodolfo Saracci

**Affiliations:** European Educational Programme in Epidemiology, Florence, Italy

**Keywords:** displaced people health, refugees health, migrants health, public health challenges, displaced people epidemiology

This editorial provides the context for an initiative led and funded by the European Educational Programme in Epidemiology (EEPE) in collaboration with the Swiss School of Public Health (SSPH+). It consist of a **call for papers addressing health challenges of displaced people and a related competition for the “Rodolfo Saracci Best Paper Award 2025”** .

Apparently nobody wants to know that contemporary history has created a new kind of human beings—the kind that are put in concentration camps by their foes and in internment camps by their friends. (Hannah Arendt, 1943) [1]

Displaced persons can be defined in accordance with the International Office for Migration (IOM) [2] as: “persons who have been forced or obliged to flee or to leave their homes or places of habitual residence, in particular as a result of or in order to avoid the effects of armed conflict, situations of generalized violence, violations of human rights or natural or human-made disasters. This definition covers both internal and cross-border displacement.” They include a spectrum of more specifically defined populations such as migrants, refugees, asylums seekers, etc., with the common trait being forced displacement from the home or place of habitual residence. Displaced populations are not occasional, minor groups; they represent a sizable structural aspect of contemporary world demography [3], having increased from about 20 million in 2000 to about 110 million in 2023 (more than the combined populations of Italy and Spain), of which 43.3 million are children and adolescents below age 18. The largest fraction (62.5 million) is composed of people forcibly displaced within a country, while 36.4 million are refugees, 5.3 million asylum-seekers and 5.3 million other people in need of international protection. These are conservative estimates because of difficulties in tracing and registering people on the move.

Basic demographic data on displaced people can be found in reports of specialized national, international or non-governmental organizations. The IOM [2] documents the hardest negative health endpoint, mortality, for “in transit” displaced people on several world routes, including the ones of prominent relevance for Europe, i.e., across the Mediterranean Sea. Over 10 years (2014–2023) more than 1,800,000 attempted passages were recorded involving 28,900 deaths (1,168 of children), more than 90% by drowning. Even elementary analyses of such data are informative. As an example, it could be noted that the Italian policies to hinder migration did reduce the number of arrivals to Italy by the Central Mediterranean route over the period 2014–2018 (a result proudly claimed by the government), but at the same time trebled the risk of drowning [4], making this route the deadliest way to Europe.

Investigations of the health and disease determinants involve three groups of potential hazards. *At the place of departure*, different factors, such as environmental catastrophes, wars or civil unrest, act to force the move and at the same time can affect health. *At the displacement*, i.e., during a “transit time” lasting from hours to years, people are exposed to hazardous travel conditions, aggressions, temporary arrest often under duress. *At the arrival point*—which may sometimes be only a stopover to further displacements (a current extreme case is the fast succession of forced displacements of the Gaza population)—they may experience stressful environmental and residential conditions, inadequate food, or hostile social surroundings. Children, an important component of displaced populations, are bound to bear the full impact of exposure to these hazards more than anybody and throughout adult life [5]. A substantial volume of research on such determinants in migrants generally, as reviewed in 2018 by the UCL-Lancet Commission on Migration and Health [6], is often relevant to forced migrants as well. Papers reporting research on this particular group numbered an average of 892 in 2020–2023 (up from 380 in 2010–2013) under the PubMed [7] heading “displaced people health” and 1,411, up from 317, under the heading “refugees health.” An excellent example [8] of the most needed type of study, contributing to both scientific understanding and policy guidance, provided evidence disproving the widely disseminated “pull factor” claim that the availability of search-and-rescue boats induces migration.

Today’s contemporary history, whilst profoundly different in so many respects from Hannah Arendt’s [1], echoes it by continuously generating forcibly displaced people, often deprived of “the right to rights” and undergoing endless hardship in camps. Forces driving people out of their homes are on the increase, particularly climate deregulation and ongoing wars (90 in 2010, 180 in 2022) [9] and a projection puts the additional figure of displaced people by the end of 2025 at nearly seven million, mostly in lower income countries with fragile governance [10]. Epidemiological investigations directly geared to public health actions need to be expanded to characterize the health status of the increasing number of displaced people, clarify the pathogenic role of a variety of hazardous factors and, relatedly, identify circumstances and interventions favouring or hampering the delivery of health services. Young epidemiologists should be encouraged toward field training to gain first-hand knowledge of forced migrants’ lives and direct experience of pertinent data collection.

As witnessed by the new Pact on Migration and Asylum of the European Union [11] governments’ policies towards displaced people are mostly based on security, barriers to entry and border control, reflecting public opinions pervasively influenced by nationalist ideologues, while health is lost to sight (I came across, only once, the words “medical care”). Hostile attitudes and defensive policies cannot be thwarted solely by epidemiological documentation and investigations of the conditions and health of placeless people: but without these, Arendt’s “nobody wants to know” will only grow.
